# Race and rehabilitation following spinal cord injury: equality of access for American Indians/Alaska Natives compared to other racial groups

**DOI:** 10.1186/s40621-015-0049-0

**Published:** 2015-07-07

**Authors:** Alan D Cook, Jeanette G Ward, Kristina M Chapple, Hassan Akinbiyi, Mark Garrett, Forrest O’Dell Moore

**Affiliations:** Chandler Regional Medical Center, Chandler, AZ USA

## Abstract

**Background:**

Representing 2 % of the general population, American Indians/Alaska Natives (AIs/ANs) were associated with 0.5 % (63) of the estimated 12,500 new cases of spinal cord injury (SCI) reported to the National Spinal Cord Injury Statistic Center in 2013. To date, the trend in health care disparities among AIs/ANs in the SCI community has not been examined. We sought to compare the rate of discharge to rehabilitation facilities (DRF) following traumatic SCI among adult AIs/ANs to other racial/ethnic groups for patients 15 to 64 years old.

**Methods:**

Utilizing data from the National Trauma Data Bank (NTDB), we performed a retrospective analysis of SCI cases occurring between January 1, 2008 and December 31, 2012. SCI injuries were identified by International Classification of Diseases 9th Revision-Clinical Modification (ICD-9) codes or Abbreviated Injury Scale (AIS) scores. Injury severity was determined using the Trauma Mortality Prediction Model (TMPM) which empirically estimates each patient’s probability of death given their individual complement of injuries. A series of seven logistic regression models were used to predict DRF between racial groups.

**Results:**

Among the 29,443 patients in our cohort, 52.4 % were discharged to rehabilitation facilities. AIs/ANs comprised 1.1 % of the population, with 63.8 % dismissed to rehabilitation. AIs/ANs were significantly younger, had a higher probability of death, had longer hospital length of stay (HLOS), and were proportionately more likely to be discharged to rehabilitation compared to non-AIs. Regression models demonstrated increased odds of DRF for AIs/ANs compared to Hispanic and Asian racial/ethnic groups.

**Conclusions:**

American Indians/Alaska Natives who sustain SCI access rehabilitative care at a rate equitable to or greater than other races when multiple factors are taken into account. Further research is needed to assess the effect of those patient, physician, and health care system determinants as they relate to a patient’s ability to access post-trauma rehabilitative care. Recommendations include advancing the level of racial, insurance, and geographic data necessary to adequately explore disparities related to such ubiquitously life-altering conditions as SCI.

## Background

In the USA, an estimated 12,500 new cases of spinal cord injury (SCI) are reported to the National Spinal Cord Injury Statistic Center each year (NSCISC 2014). SCI is most commonly the result of a motor vehicle crash and has been reported as the neurologic injury resulting in the highest need for rehabilitation (Office of Management and Budget 1997). A consensus exists regarding the need for additional research to disentangle the effects of minority status on functional outcomes and need for rehabilitative care following SCI. This is evidenced by an increased risk of secondary complications, decreased quality of life, and increased depressive symptomatology when SCI patients are not treated by rehabilitative services (Lad et al. 2013; Saladin and Krause 2009; Cardenas et al. 2004; Krause et al. 2006, 2009; Myaskovsky et al. 2011; Hunt et al. 2004; Ambrosio et al. 2007).

Minorities including the American Indian/Alaska Native (AI/AN) population have been reported to experience disparate levels of functional independence during inpatient rehabilitation and longer rehabilitation length of stay, and to be at higher risk of discharge to home versus rehabilitation (Burnett 2002). Among minorities identified as being at highest risk for a particular type of traumatic injury or mechanism, it is possible that literature has obscured or removed subpopulations prior to analysis due to their smaller sample size by aggregating smaller racial/ethnic groups into a single category, e.g., “other race.” In 2000, Burhansstipanov and Satter presented an overview of the AI/AN population, noting that the routine collapsing of smaller minority populations into an “other” category or excluding them altogether during analysis can be detrimental to providing accurate results (Burhansstipanov 2000). Policymakers, researchers, and AI/AN tribal planners require such evidence to develop effective programs with which to aid the AI/AN population. The Office of Management and Budget (OMB) recommends all federally funded research and service projects follow the racial categories outlined in Directive 15 when reporting study findings (Burhansstipanov 2000). These include American Indian or Alaska Native, Asian, Black or African American, Native Hawaiian or Other Pacific Islander, and White. There are two categories for data on ethnicity: “Hispanic or Latino” and “Not Hispanic or Latino” (Office of Management and Budget 1997).

The AI/AN population currently represents approximately 2 % of the total United States (U.S.) population and is noted to be one of the fastest growing racial minorities (United States Census Bureau 2010). In 2010, approximately 2.6 million people identified their race as AI/AN alone, whereas 5.2 million identified themselves as AI/AN in combination with one or more other races (United States Census Bureau 2010). This minority’s growth rate is currently three times that of the national rate (27 versus 9 %) (United States Census Bureau 2010). When compared to other Americans, AIs/ANs are nearly 3 times more likely to experience unintentional traumatic injury and 3.4 times more likely to experience a motor vehicle crash (Indian Health Services 2014; Pollack et al. 2012). Sixty-three (0.5 %) of the 12,500 new SCI patients reported by the NSCISC are sustained by AIs/ANs (NSCISC 2014). However, among minorities, the rate of discharge to rehabilitation facilities (DRF) following SCI remains infrequently and unevenly reported despite AI/AN heritage having emerged as a predictor of discharge to a nursing home versus rehabilitative care (DeVivo and Fine 1999). Few researchers who have studied the topic of DRF have included the AI/AN population in their analysis (Krause and Broderick 2004; Krause et al. 2009, 2014; Gary et al. 2011; Lad et al. 2013; Mansfield et al. 2014).

Regardless of race, unmet need for DRF is often determined by a combination of non-mutually exclusive variables such as injury severity, reimbursement patterns, and type of insurance. Various factors (individual, provider, and health care system/policy) may prove to be strong predictors for DRF following SCI. As such, we sought to elucidate the rate of DRF following traumatic SCI among adult AIs/ANs (15 to 64 years of age) compared to other racial/ethnic groups when various important factors are taken into account. We hypothesized that DRF following SCI will differ negatively for AIs/ANs compared to other racial/ethnic groups.

## Methods

We performed a cross-sectional retrospective analysis of data from the National Trauma Data Bank (NTDB). Developed by the American College of Surgeons (ACS) in 1989, the NTDB is the largest aggregation of voluntarily submitted, nationally representative trauma registry data (ACS 2014). To guarantee anonymity, NTDB data are purged of all identifying patient and hospital identifiers. Currently, the NTDB contains detailed data on over five million cases from over 900 registered U.S. trauma centers (ACS 2014). Data are voluntarily submitted by participating hospitals. Details describing the methodology behind various NTDB data collection procedures have been extensively described elsewhere (Haider et al. 2012; Sperry et al. 2012; Branco et al. 2011; Bukur et al. 2012; Sullivent et al. 2011). This study was approved by the Institutional Review Board of the Chandler Regional Medical Center, Chandler, Arizona.

SCI cases occurring between January 1, 2008 and December 31, 2012 were identified among over three million patient records using International Classification of Diseases, 9th Revision-Clinical Modification (ICD-9) diagnosis codes from the 806.xx and 952.xx series and Abbreviated Injury Severity codes 6306XX and 640XXX. Injury severity was determined using the Trauma Mortality Prediction Model (TMPM) that estimates each patient’s probability of death based on their individual combination of injuries (Glance et al. 2009). In the NTDB, DRF indicates that all insurance-related pre-authorizations have been obtained prior to hospital dismissal. DRF was defined as discharge to either an acute rehabilitation unit or a skilled nursing facility. The cohort included patients aged 15 to 64 years old. We included all racial/ethnic categories represented in the NTDB registry: Asian, Native Hawaiian or Other Pacific Islander, Other race, American Indian or Alaska Native, Black or African American, Hispanic, and White. Hispanic ethnicity included members of these racial categories who classified themselves as such. Racial/ethnic classifications were based on patient self-report. Exclusionary criteria included patients (1) declared dead on arrival, who died in the hospital, or were dismissed to hospice; (2) dismissed to home from the emergency department; (3) with unknown race, age, insurance type, or discharge destination; (4) who sustained burn injuries; and (5) who were seen at facilities which did not report any AI/AN among hospital admissions during the study period. These facilities were, by definition, excluded from the cohort as they were unable to dismiss AI/AN patients to a rehabilitation center. The primary outcome was DRF versus other discharge disposition.

### Statistical analysis

Frequencies with percentages, and means with standard deviations, were used to describe the overall cohort of SCI patients. Independent sample *t*-tests were used to compare mean differences for continuous parametric variables. Chi-square tests were used to compare distributions for ordinal or dichotomous variables. Partial eta-squared values are reported to demonstrate effect size. For the purposes of the univariate analysis, a dichotomous variable was created (i.e., rehabilitation service) to demonstrate patients dismissed to either a rehabilitation facility or a skilled nursing unit. A series of hierarchical multivariate logistic regression (HMVLR) models clustered on hospital facility and used to predict hospital DRF from race were then performed. Covariates in the model included age and gender, insurance (payment), facility volume, treatment complications, hospital length of stay (HLOS), and injury severity. The first model compares the AI/AN group to all non-AIs/ANs. Subsequent models compared the AI/AN group to each of the individual racial groups: White, Black or African American, Hispanic, Asian, Hawaiian or Pacific Islander, and Other. For the purposes of the models, the variable payer was coded such that a response of uninsured was utilized as the reference group and compared to the privately insured (i.e., insurance provided by one’s employer), government insurance (i.e., Medicaid, Medicare, Veterans Benefits Administration, Indian Health Services), and payment (other) cohorts. A variable based on facility volume was also created. The top quartile of cases based on facility volume was designated as “high volume” and included in the regression models. The most frequently occurring hospital complications were also included in the regression models. Predicted probabilities from models were used to calculate the area under the receiver operating characteristic curve. The continuous variables of HLOS and probability of death were logarithmically transformed to account for skew and kurtosis. To estimate bias due to missing data, we first tested whether the missing data were missing completely at random by applying a user-written routine for Stata, ~mcartest~. Our missing data were not missing completely at random. Next we assessed whether the missingness in our dataset biased our results. An HMVLR model was developed using the multiple imputation suite of commands in Stata 14.0. No significant difference was found between the imputed models and the model shown. Model discrimination was estimated using the area under the receiver operating characteristic (AUC) curve (Hosmer et al. 2013). SPSS version 22 and Stata version 14.0 were used for statistical analysis. *P* values of less than 0.05 were considered statistically significant.

## Results

Following application of exclusion criteria and deletion of cases with missing variables (see Tables 5 and 6 for a tabulation of exclusion criteria), our complete cohort consisted of 29,433 patients. The largest proportion of excluded patients represented patient age outside of the 15 to 64 group of interest. The second highest exclusion criterion was those treatment hospitals that did not discharge a single AI/AN during the study period (2008 to 2012). To confirm that our results remained static if all facilities were included, we performed a sensitivity analysis by running our models on the larger dataset without the excluded group of hospitals and obtained comparable results.

Cohort characteristics are shown in Table 1. AIs/ANs represented 1.1 % (*n* = 318) of the cohort. Fifty-two percent (*n* = 15,417) of the cohort was discharged to rehabilitation services. American Indians/Alaska Natives were significantly younger (35.9 years ± 12.8 versus 38.3 years ± 14.5; *P* = 0.001), had a higher severity of injury (probability of death) (0.14 ± 0.15 versus 0.11 ± 0.15; *P* = 0.001), and had longer HLOS (17.3 days ± 17.5 versus 14.4 days ± 18.2; *P* = 0.004) compared to non-AIs/ANs.

**Table 1:** Cohort characteristics

Parameter	Entire sample	AI/AN	Non-AI/AN	*P* value
Age (years)	38.3 ± 14.4 (range 15–64)	35.9 ± 12.8	38.3 ± 14.5	0.001
Gender (male)	22,606 (76.8 %)	224 (70.4 %)	22,382 (76.9 %)	<0.001
Race				
American Indian/Alaska Native	318 (1.1 %)			
White	18,423 (62.6 %)			
Black or African American	5600 (19.0 %)			
Asian	572 (1.9 %)			
Native Hawaiian or Pacific Islander	114 (0.4 %)			
Hispanic	3700 (12.6 %)			
Other	706 (2.4 %)			
Insurance				<0.001
Private insurance	14,764 (50.2 %)	94 (29.6 %)	14,670 (50.4 %)	
Uninsured	5671 (19.3 %)	50 (15.7 %)	5621 (19.3 %)	
Medicaid	4926 (16.7 %)	93 (29.2 %)	4833 (16.6 %)	
Medicare	1355 (4.6 %)	13 (4.1 %)	1342 (4.6 %)	
Other	1582 (5.4 %)	13 (4.1 %)	1569 (5.4 %)	
Other government	1135 (3.9 %)	55 (17.3 %)	1080 (3.7 %)	
Rehabilitation service (Y)	15,417 (52.4 %)	203 (63.8 %)	15,214 (52.3 %)	<0.001
Probability of death	0.11 ± 0.15 (range 0.00–0.98)	0.14 ± 0.15	0.11 ± 0.15	0.001
HLOS (days)	14.4 ± 18.2 (range 1–314)	17.3 ± 17.5	14.4 ± 18.2	0.004
ICULOS (days)	10.3 ± 13.3 (range 1–314)	11.6 ± 11.9	10.3 ± 13.3	0.109
Ventilation (days)	14.0 ± 17.0 (range 1–219)	13.9 ± 13.7	14.0 ± 17.1	0.970
Discharge destination				0.001
Rehabilitation, long-term care	13,866 (47.1 %)	179 (55.9 %)	13,687 (47.0 %)	
Home without services	11,884 (40.4 %)	100 (31.4 %)	11,784 (40.5 %)	
Home with services	886 (3.0 %)	3 (0.9 %)	883 (3.0 %)	
Skilled nursing facility	1551 (5.3 %)	25 (7.9 %)	1526 (5.2 %)	
Transfer to short-term general hospital	856 (2.9 %)	10 (3.1 %)	846 (2.9 %)	
Transfer to acute care center	211 (0.7 %)	2 (0.6 %)	209 (0.7 %)	
Left against medical advice	179 (0.7 %)	0	179 (0.7 %)	

Mean ± SD or count (%)

Univariate comparisons of characteristics reported by patients who received versus did not receive rehabilitation services at discharge are shown in Table 2. Those discharged to rehabilitation demonstrated significantly higher means with regard to age, HLOS, intensive care unit length of stay (ICULOS), days on mechanical ventilator, and probability of death (*P* < 0.001), but with small effect sizes. More males (53.8 %) were dismissed to rehabilitation, whereas more females (52.2 %) failed to receive rehabilitation, *P* < 0.001. Proportionately, more AIs/ANs were discharged to rehabilitation compared to non-AIs/ANs (odds ratio (OR) 1.61, 95 % CI 1.28–2.03). Depending on the insurance provider, DRF ranged from 40.9 to 62.6 %, *P* < 0.001.

**Table 2:** Discharge to rehabilitation by patient characteristics

Parameter	Rehabilitation (no) (*n* = 14,016)	Rehabilitation (yes) (*n* = 15,417)	*P* value	Effect size or odds ratio (95 % confidence interval)
Age (years)	37.8 ± 14.1	38.7 ± 14.7	<0.001	0.001
HLOS (days)	8.1 ± 13.6	20.1 ± 19.9	<0.001	0.108
ICULOS (days)	5.9 ± 9.7	12.3 ± 14.2	<0.001	0.050
Ventilation (days)	10.1 ± 16.5	15.0 ± 17.0	<0.001	0.014
Probability of death	0.1 ± 0.1	0.2 ± 0.2	<0.001	0.075
Gender			<0.001	1.27 (1.20–1.34)
Female	3559 (52.2 %)	3265 (47.8 %)		
Male	10,454 (46.2 %)	12,152 (53.8 %)		
Race			<0.001	
American Indian/Alaska Native	115 (36.2 %)	203 (63.8 %)		
White	8740 (47.4 %)	9683 (52.6 %)		
Black or African American	2274 (40.6 %)	3326 (59.4 %)		
Asian	315 (55.1 %)	257 (44.9 %)		
Native Hawaiian or Pacific Islander	59 (51.8 %)	55 (48.2 %)		
Hispanic	2159 (58.4)	1541 (41.6 %)		
Other	354 (50.1 %)	352 (49.9 %)		
Race			<0.001	1.61 (1.28–2.03)
American Indian/Alaska Native	115 (36.2 %)	203 (63.8 %)		
Non-American Indian/Alaska Native	13,901 (47.7 %)	15,214 (52.3 %)		
Insurance			<0.001	
Private	6885 (46.6 %)	7879 (53.4 %)		
Uninsured	3350 (58.9 %)	2321 (40.9 %)		
Medicaid	1842 (37.4 %)	3084 (62.6 %)		
Medicare	555 (41.0 %)	800 (59.0 %)		
Other government	640 (56.4 %)	495 (43.6 %)		
Other	744 (47.0 %)	838 (53.0 %)		

Associations between patient complications and comorbidities with DRF were examined (data not shown). The most frequent patient complications were pneumonia (*n* = 3556, 12.1 %), acute respiratory distress syndrome (*n* = 1526, 5.2 %), pressure ulcer (*n* = 1441, 4.9 %), and deep vein thrombosis (*n* = 1098, 3.7 %). The presence of each of the listed complications was associated with increased odds of DRF with ORs ranging from 4.8 for acute respiratory distress syndrome to 6.4 for pressure ulcer. The most frequently reported patient comorbidities were smoking (*n* = 4444, 15.1 %), hypertension requiring medication (*n* = 4298, 14.6 %), alcoholism (*n* = 3909, 13.3 %), and diabetes mellitus (*n* = 1895, 6.4 %). Although patient comorbidities were more commonly reported than were complications, the strength of association with our outcome variable was modest with ORs ranging from 1.1 for alcoholism to 1.3 for diabetes mellitus.

### Regression models

A series of HMVLR models were used to understand the interplay of patient and facility characteristics as predictors of DRF. Results from all seven models are shown in Tables 3 and 4 with *P* values, ORs, and confidence intervals. The AUC was above 0.80 in all models, suggesting the models were strong discriminators of hospital discharge to rehabilitation. Depending on the model, intraclass correlation coefficients suggested that clustering on hospital facility accounted for 12 to 21 % of the variability in hospital DRF.

**Table 3:** AI versus all non-AI/AN

Parameter	*P* value	OR (95 % CI)
Age	0.062	
Gender (male)	0.269	
High-volume facility	0.740	
Racial comparison	0.790	
Complication: pressure ulcer	0.197	
Complication: acute respiratory distress syndrome (ARDS)	0.078	
Complication: deep vein thrombosis (DVT)	0.037	1.23 (1.01–1.48)
Complication: pneumonia	0.190	
Payment (reference: uninsured)		
Private insurance	0.000	2.21 (2.03–2.40)
Government insurance	0.000	2.15 (1.96–2.37)
Payment, other	0.000	1.77 (1.51–2.08)
Hospital length of stay (log)	0.000	32.07 (28.71–35.82)
Probability, death (log)	0.000	1.40 (1.31–1.49)
Sample size	29,426	
Area under the curve (AUC)	0.86 (0.86–0.87)

**Table 4:** HMVLR models comparing American Indians/Alaska Natives to other racial groups for predicting discharge to rehabilitation

	AI/AN versus White		AI/AN versus African American		AI/AN versus Hispanic	
	*P* value	OR (95 % CI)	*P* value	OR (95 % CI)	*P* value	OR (95 % CI)
Age	0.059		0.028	0.99 (0.99–1.00)	0.479	
Gender (male)	0.260		0.393		0.478	
High-volume Facility	0.614		0.949		0.907	
Racial comparison	0.976		0.659		0.010	1.61 (1.12–2.32)
Complication: pressure ulcer	0.457		0.002	0.56 (0.39–0.81)	0.498	
Complication: ARDS	0.002	0.72 (0.58–0.89)	0.390		0.095	
Complication: DVT	0.832		0.002	2.32 (1.37–3.94)	0.100	
Complication: pneumonia	0.174		0.625		0.686	
Payment (reference: uninsured)						
Private insurance	0.000	2.43 (2.17–2.73)	0.000	1.63 (1.35–1.95)	0.000	2.47 (1.95–3.13)
Government insurance	0.000	2.26 (1.97–2.58)	0.000	1.89 (1.58–2.27)	0.000	2.24 (1.75–2.87)
Payment, other	0.000	2.04 (1.64–2.52)	0.024	1.48 (1.05–2.07)	0.007	1.70 (1.16–2.51)
Hospital length of stay (log)	0.000	50.79 (43.79–58.90)	0.000	22.30 (17.59–28.27)	0.000	15.21 (11.48–20.16)
Probability, death (log)	0.000	1.45 (1.33–1.57)	0.001	1.29 (1.12–1.50)	0.000	1.41 (1.18–1.67)
Sample size	18,736		5916		4017	
Area under the curve (95% CI)	0.87 (0.87–0.88)		0.86 (0.85–0.87)		0.88 (0.87–0.89)	
	AI/AN versus Asian		AI/AN versus Hawaiian Pacific Is.		AI/AN versus Other	
	*P* value	OR (95 % CI)	*P* value	OR (95 % CI)	*P* value	OR (95 % CI)
Age	0.268		0.875		0.744	
Gender (male)	0.950		0.692		0.217	
High-volume facility	0.822		0.207		0.013	0.59 (0.39–0.89)
Racial comparison	0.026	1.66 (1.06–2.59)	0.747		0.075	
Complication: pressure ulcer	0.643		0.953		0.884	
Complication: ARDS	0.034	0.31 (0.11–0.92)	0.003	0.11 (0.03–0.48)	0.170	
Complication: DVT	0.753		0.283		0.887	
Complication: pneumonia	0.459		0.933		0.608	
Payment (reference: uninsured)						
Private insurance	0.000	3.00 (1.71–5.26)	0.636		0.005	1.93 (1.22–3.05)
Government insurance	0.053		0.283		0.035	1.67 (1.04–2.71)
Payment, other	0.203		0.748		0.602	
Hospital length of stay (log)	0.000	49.70 (25.59–96.52)	0.000	110.39 (35.85–339.92)	0.000	18.08 (10.77–30.35)
Probability, death (log)	0.028	1.54 (1.05–2.62)	0.617		0.450	
Sample size	889		431		1022	
Area under the curve (95% CI)	0.88 (0.86–0.90)		0.91 (0.88–0.94)		0.85 (0.83–0.88)	

Race, the predictor of highest interest to our study, was predictive of DRF in two of seven models: AI/AN versus Hispanic and Asian with the AI/AN group demonstrating increased odds of 1.61 (95 % CI 1.12–2.32) and 1.66 (95 % CI 1.06–2.59) times more in favor of the AI/AN group, respectively. There was no statistically significant difference in DRF between the AI/AN group and all other races combined (Table 3), or when compared to Whites, Blacks or African Americans, Hawaiian/Pacific Islanders, or Other races (Table 4).

The importance of covariates varied across models. Hospital length of stay, probability of death, and payer were significant covariates in nearly every model, whereas gender was not significant in any model. Patient age was a significant covariate in one model (AI/AN versus Black or African American) but with a *P* value of just less than 0.05 and a confidence interval with an upper limit rounded to 1.0. Hospital complications emerged as significant covariates, but sporadically. Across all models, four of eight significant complications were negatively associated with DRF. Pneumonia was the most common complication with more than twice as many cases in comparison to the second most common complication, but did not emerge as a significant covariate in any of the seven models.

## Discussion

In 2002, the Institute of Medicine issued a call to action to eliminate racial and ethnic health disparities (Institute of Medicine of the National Academies 2002). To ensure optimum health outcomes for indigenous peoples following acute SCI, it is important to fully appreciate their ability to access prescribed rehabilitative care. DRF not only is a critical component in patients achieving their full recovery potential, but also answers the Institute of Medicine’s call.

Heretofore, the exclusion of the AI/AN race due to sample size, the amalgamation of all smaller minorities into an aggregate “Other” group, and the recognized effect of racial misclassification may have obscured the number of AIs/ANs within any given sample (Office of Management and Budget 2000). In the presence of ongoing controversy regarding ill-defined racial categories in medical research, dialogue is actively encouraged by the editors of leading medical and scientific journals (Council of Biology Editors, Style Manual Committee 1994; United States Census Bureau 2000; Bhopal and Donaldson 1998; Schwartz 2001; Wood 2000; Editor 2001; Burchard et al. 2003; Cooper 2003; Karter 2003; Ellison 2005; Braun 2006). The OMB recommends summary studies comply with recommendations to independently assess small numbers of selected racial groups (Burhansstipanov 2000). The U.S. Census Bureau projects non-regionally specific growth within the AI/AN community to be double that of the general U.S. population in 2010 (Norris et al. 2010). As such, the need to understand outcome patterns for this minority is urgent.

Overall, our cohort was similar to previous trauma-driven racial disparity studies with regard to median age, gender, and incidence rate of SCI (NSCISC 2014). In examining the most recent 5 years of NTDB data, we failed to identify a negative disparity in DRF for AI/AN patients with SCI compared to other racial groups when other factors were taken into account.

However, univariate comparison of AIs/ANs to other racial groups demonstrated differences in age, gender, insurance type, HLOS, severity of injury (probability of death), and discharge destination. Multiple patient demographics, as well as injury characteristics, were controlled in our models to determine the independent effect of racial classification of rates of DRF.

Although similarities were noted for age, gender, and incidence, our findings contrasted with literature from other health care domains which describe lower rates of access to rehabilitation across a wide range of illnesses (Centers for Disease Control and Prevention 2007; Chien et al. 2007; Pierce 2007; Smedley et al. 2002). We report AIs/ANs received rehabilitation at an equitable rate with other races with the exception of the Hispanic/Latino and Asian groups. These findings support those of Shafi et al., who reported equivalent access to acute rehabilitation among minorities. However, Shafi’s paper excluded AIs/ANs from the analysis (Shafi et al. 2007).

In 2013, Lad et al. investigated the presence of racial disparities on HLOS, complications, and hospital disposition following non-isolated SCIs from the NTDB 2000 to 2009 (Lad et al. 2013). Whereas Lad’s findings add key evidence to the understanding of DRF patterns among minority patient populations, differences in dismissal patterns between the AI/AN population versus all other races were not explored. In a departure from the more commonplace practice of collapsing small minority populations prior to analysis, like Lad, we looked at all seven independent racial categories available within the NTDB. Methodological differences included Lad’s comparison of all minorities to Whites, whereas we compared all other races to the AI/AN population. With regard to admission to an acute rehabilitation center, Lad et al. reported no difference between Whites and AIs/ANs. In the present study, however, differences emerged in comparisons of AIs/ANs to both Hispanics (OR = 1.61) and Asians (OR = 1.66). Our findings were consistent with regard to DRF comparing AIs/ANs to Whites. Lad also reported a significant difference for gender between racial groups; however, although statistically significant on univariate analysis, gender played no role in predicting DRF in our models regardless of racial comparison model.

Beyond racial classification, the remainder of the variables tested in our models was unstable, with significance and direction of effect varying by model. Our findings generally suggest higher DRF for patients with any type of insurance. However, odds ratios were modest and did not exceed 3.0 in any model. Fifty percent of our AI/AN population were noted to carry Medicare, Medicaid, or other government insurance (which includes Indian Health Service (IHS)) at discharge, approximately twice that of our non-AI/AN group. This was surprising, given reports that approximately one in three AIs/ANs under the age of 65 is uninsured (Centers for Disease Control and Prevention 2012). We found 50 % of non-AIs/ANs had access to private insurance, compared to 30 % of AIs/ANs; however, we were unable to delineate those AIs/ANs with access to both private insurance and IHS (government) insurance. Although insurance is sometimes used as a surrogate for socioeconomic status, no data are available in the NTDB to allow us to substantiate such associations.

Whereas Lad’s study demonstrated AIs/ANs had the highest odds of pneumonia and deep vein thrombosis (DVT)/PE, and odds second only to Native Hawaiians for pressure ulcer, our complications and comorbidity results were scattered. Longer HLOS for AIs/ANs is potentially associated with the length of time necessary to receive IHS authorization for discharge to rehabilitation or high rates of diabetes mellitus, a baseline health condition associated with health disparities and shown to prolong recovery times (Kao et al. 2006; Indian Health Service 2014). In addition, AIs/ANs are 1.6 times more likely than either Whites or Hispanics to experience in-hospital complications (Lad et al. 2013). Respiratory problems, pressure sores, and urinary tract infections are common post-SCI complications (Gary et al. 2011). The presence of pressure ulcer has been reported to be highly correlated with an AI/AN racial classification following SCI (Braun 2006). Waites et al. reported AIs/ANs and African Americans faced a higher risk of developing either a UTI or pressure ulcer following SCI (Waites et al. 1993). Our findings support those reported by Lad et al., where AIs/ANs were found to have proportionally longer HLOS.

In 1999, DeVivo et al. reported American Indian/Alaska Native heritage as a strong predictor for discharge to a nursing home (DeVivo and Fine 1999). Using NTDB data, Englum et al. reported a 5.0 % rate of DRF and 5.4 % to nursing facilities; however, the AI/AN minority group was excluded from their analysis (Englum et al. 2011). For our AI/AN group, we report a 63.8 % rate of DRF. Hospitals with increased volume saw the most serious cases and discharged more patients to rehabilitation. One speculative explanation is higher hospital volume and overall injury severity may represent mature systems of trauma care that include the availability of rehabilitation services.

Despite the oftentimes limited amount of data available, the past three decades have seen significant advances in attempts to elucidate explanations for racial disparities in health care outcomes. A percentage of these determinants are rooted in causes associated with patients, providers, and health care systems. Of particular importance is the shift in the demographics of SCI patients since 1990, where risk has increased for minority patients and now eclipses that of Whites. Understanding the implications of obtaining a full health history inclusive of race, comorbidities, and patient’s ability to synthesize medical information is a key consideration for physicians in the development of appropriate treatments and ultimately their ability to improve their patient’s quality of life. This and other similar studies are targeted attempts to explain and lower the level of racial disparities in the trauma population and inform future hypotheses.

We note that pressure ulcer and acute respiratory distress syndrome were associated with decreased odds of DRF as these events potentially portend poorer prognoses leading to discharge to facilities other than acute rehabilitation centers. This topic is beyond the scope of this study, however.

### Limitations

This study has several limitations. The NTDB is a convenience sample, and as such, generalizability to the entire trauma patient population cannot be assumed as the data represent only those patients (minority and non-minority) with access to trauma centers who submit data to the NTDB and therefore does not include all SCI sustained in either population. The NTDB includes data from a disproportionate number of larger hospitals with younger and more severely injured patients. The data may not be representative of all trauma hospitals in the nation and thus may not allow statistically valid inferences about national injury incidence and prevalence (American College of Surgeons 2014). Moreover, our study included only those centers that reported one or more AIs/ANs after other exclusions were applied. In addition, the NTDB files contain administrative and trauma registry data, which may differ in terms of data completeness and accuracy between facilities. Therefore, these data were not gathered for the purpose of studying race, ethnicity, or the association of either of these with patient discharge to rehabilitation facilities.

The potential exists for misclassification of race/ethnicity in our cohort due to possible information bias via variation in data gathering practices, i.e., self-identification versus racial classification by hospital staff. The Affordable Care Act provides guidelines to strengthen the validity and reliability of administratively collected racial data (U.S. Government Publishing Office 2010). Although the NTDB does not currently provide the necessary level of granularity to adequately explore racial misclassification, continued improvement in data integrity is expected to emerge following implementation of the Affordable Care Act.

Our results may be also biased by our exclusion criteria. The individual and cumulative frequencies of the exclusion criteria are shown in Tables 5 and 6. As data integrity, including missing values and correct coding of key variables, is an area of concern with large databases, we performed a sensitivity analysis by running our models on a larger dataset without those cases excluded in the final analysis and obtained similar where race remained predictive in two of our seven models.

**Table 5:** Exclusion criteria among 61,517 patients with SCI in the NTDB 2008 to 2012

Exclusion criteria	Count (% of 61,517)
Age not between 15 to 64 years	13,964 (22.7 %)
No AIs/ANs dismissed to rehabilitation	8323 (13.5 %)
Patient not admitted	8228 (13.4 %)
Insurance status unknown	7082 (11.5 %)
Discharge destination unknown	6931 (11.3 %)
Death in hospital	4529 (7.4 %)
Race unknown	2144 (3.5 %)
Exclusions ICD-9	219 (0.4 %)
Discharged to hospice	158 (0.3 %)

**Table 6:** Sum of exclusion criteria

Number of exclusions	Number of cases	Percent	Cumulative percent
0	29,433	47.8	47.8
1	18,104	29.4	77.3
2	9435	15.3	92.6
3	3686	6.0	98.6
4	764	1.2	99.8
5	81	0.1	100.0
6	13	0.0	100.0
7	1	0.0	100.0
Total	61,517	100.0	

In addition, as the inclusion of hospitals that did not discharge AIs/ANs potentially impacted our analyses, we reran our models using a larger sample size which included these excluded facilities. Findings from the seven models were consistent with our reported models with increased odds of DRF for AIs/ANs in comparison to Hispanics and Asians. An in-depth investigation of bias due to missing or misclassified data is beyond the scope of this study.

Findings included various trauma hospital levels. ACS trauma hospital accreditation levels (i.e., levels I, II, III, and IV) refer to the level of trauma care capability available at a given facility and do not imply uniformity of data submission. Trauma centers are disproportionately distributed throughout the USA, potentially producing skew when assessing hospital admissions following SCI, and subsequently DRF for minorities and non-minorities alike. In addition, the ACS reports hospitals respond in a disproportionate manner to the annual Call for Data, determined by calculating the ratio of hospitals who respond to those eligible to submit data (ACS 2014).

No data are available to differentiate between patients who arrived with insurance from those who qualified for government insurance prior to dismissal. Thus, insurance in place on admission cannot be extrapolated as a measure of socioeconomic status. Lack of facility details may have led to a homogenous view of rehabilitation facilities, and this oversimplification may have masked important nuances in discharge destination. Finally, the *P* values associated with our univariate models were highly significant, although effect sizes were negligible indicating our large sample was detecting very small effects that may not be clinically meaningful. Despite these limitations, this study provides a sophisticated analysis of predictors for rehabilitation specific to the AI/AN race. Clear associations between multi-level measures of race and ethnicity and use of post-hospitalization care were demonstrated.

## Conclusions

The patterns demonstrated in our study indicated that when compared to other races, those AIs/ANs who sustain a SCI access rehabilitative care at an equitable, rather than disparate, rate. This is perhaps an indication that headway is being made in lowering the unmet need of this population or that shifts in the demographic composition of the AI/AN population and type of insurance they carry may have initiated a pattern of correction of previously noted access-related disparities. Measures of racial classification at the national level will continue to play a role in understanding the patterns of DRF following SCI. To advance the generalizability of racial disparity studies, further research is needed to assess the effect of those patient, physician, and system determinants as they relate to a patient’s ability to access post-trauma rehabilitative care. Our findings may prove useful for those policymakers, researchers, and tribal leaders interested in access to care issues throughout the AI/AN nations, specifically among SCI-injured patients.

## Abbreviations

AIs/ANs: American Indians/Alaska Natives
AUC: area under the receiver operating characteristic
DRF: discharge to rehabilitation facilities
HMVLR: hierarchical multivariate logistic regression
HLOS: hospital length of stay
ICULOS: intensive care unit length of stay
ICD-9: International Classification of Diseases 9th Revision-Clinical Modification
NSCISC 2013: National Spinal Cord Injury Statistic Center each year
NTDB: National Trauma Data Bank
ORs: odds ratios
OMB: Office of Management and Budget
TMPM: Trauma Mortality Prediction Model
